# Need for streamlined use of DPP-4 inhibitors in the treatment of type 2 diabetes

**DOI:** 10.1186/s12933-016-0379-4

**Published:** 2016-04-02

**Authors:** Cristiana Vitale, Giuseppe M. C. Rosano, Krishna Prasad

**Affiliations:** Centre for Clinical & Basic Research, IRCCS San Raffaele Pisana, via della Pisana, 235, 00163 Rome, Italy; St George’s University of London, London, UK; St Thomas’ Hospital, London, UK

**Keywords:** Dipeptidyl peptidase 4 inhibitors, Cardiovascular, Diabetes mellitus, Empagliflozin, Cost-effectiveness, Regulatory, Clinical trials, Healthcare

## Abstract

Regulatory agencies request an assessment of cardiovascular safety for all “new” oral anti-diabetic drugs in order to avoid possible negative effects on cardiovascular events. Dipeptidyl peptidase 4 inhibitors have emerged as a new therapeutic alternative for the treatment of type 2 diabetes mellitus, but the several large post-marketing clinical trials have shown only a modest effect in glycaemic control and, more importantly, a neutral effect on total and cardiovascular events. Conversely a recent trial with empagliflozin, a sodium-glucose co-transporter 2 inhibitor, has shown significant effect on overall and cardiovascular mortality. Although glycaemic control is an important aspect of diabetes management, the results of the EMPA-REG outcome trial suggest that it is possible to develop anti-diabetic drugs that may exert an overall beneficial effect beyond the mere improvement of glycaemic control. While the regulatory hurdles should not be increased, there is the need for evaluation of the net clinical impact and cost effectiveness of all anti-diabetic agents. Therefore, a better collaboration among all stakeholders is needed in order to develop studies with endpoints that will be both clinically meaningful including appropriate follow-up, and economically relevant in patients with type 2 diabetes mellitus.

## Background

After the rosiglitazone saga, the main regulatory agencies (European Medicines Agencies, Food and Drug Administration) have requested an assessment of cardiovascular safety for all “new” oral anti-diabetic drugs (OADs). This was in order to avoid a situation that the supposed benefits, inferred from their effect on the surrogate endpoint of glycated haemoglobin (HbA1c), would not have been outweighed by negative effects on cardiovascular events. Dipeptidyl peptidase 4 inhibitors (DPP4i) have emerged as a new therapeutic alternative for the treatment of type 2 diabetes mellitus (T2DM). Several large post-marketing clinical trials have been now completed with the new DPP4i overall involving more than 36,000 T2DM patients at increased cardiovascular risk [1–5] showing a neutral effect of this new class on hard outcomes. The SAVOR TIMI-53, the EXAMINE and the VIVIDD studies raised initial doubts on the cardiovascular safety of DPP4i [1, 2, 5] related to the increased occurrence of heart failure (HF) [4–6]. Reassuringly, the recent TECOS study [3] showed that addition of sitagliptin to usual care did not affect the cardiovascular composite endpoints or the rates of hospitalization for HF. However, DPP4i differ from each other in their chemical structure and consequently their off target properties and this may influence their biological actions and therefore the overall clinical effect.

## Discussion

Glycaemic control is an important aspect of managing diabetes and reducing microvascular complications. However, one of the main aims of treatment of T2DM, would be to reduce events and cardiovascular risk in an effective and safe way. With several DPP4i authorised thus far and others undergoing extensive development programmes, one may wonder if there is sufficient certainty that their effect on glycaemic control translates into a sustained clinical benefit. In the post-marketing studies the comparative glucose lowering effect of the tested DPP4i to placebo was pretty small and no effect on hard end-points was observed [3].

It could be argued that treatment duration and study populations might have influenced the lack of benefits of DPP4i [7]. However, should a longer study duration or a different/larger population be needed to show a beneficial effect, by corollary, the magnitude of this effect would have been necessarily small and may be intangible. A combined analysis of the studies available with these drugs so far supports this thought given the size of the populations studied.

The direct comparisons with active glucose lowering comparators in drug-naive patients have demonstrated that DPP4i exert slightly less pronounced HbA1c reduction than metformin, suggesting a smaller effect on glycaemia, than the three oldest classes (insulin, the sulfonylureas, and the biguanides) in addition to higher costs that do not justify a widespread use [8].

The results of the large post-marketing trials with DPP4i highlight that this class of glucose lowering drugs provide limited beneficial effect in patients with T2DM and, have not provided an answer to the clinical need for OADs that are safe and potentially reduce cardiovascular complications. Therefore, approaches other than HbA1c lowering alone may be necessary in order to reduce cardiovascular events including total mortality and HF in these patients. This is especially true in the light of the recent results of the EMPA-REG outcome trial with empagliflozin belonging to another class of OADs, sodium-glucose co-transporter 2 inhibitors (STGL2i), that showed that, in 7020 patients with T2DM at high cardiovascular risk, the addition of empagliflozin to standard care significantly reduced the occurrence of cardiovascular outcomes, including HF in addition to reaching a target HbA1c of 7.8 % [9]. The results of this study suggest that this newer drug may exert an overall beneficial effect beyond the mere improvement of glycaemic control. This may be mediated through effect on lowering blood pressure and reduction in fluid overload or other as yet unidentified mechanisms, but more data form others in the class are required for confirmation. The magnitude of these results sets the bar for the development of future treatments for diabetes and makes preferential use of drugs such as DPP4i difficult to be justifiable in the absence of a meaningful clinical benefit, particularly in the context of wide use in all diabetic patients.

From a regulatory perspective, the glucose lowering effect of OADs is usually studied against placebo as add on often in combination with metformin in studies conducted for licensing purposes. An important question that might arise is: should there be a requirement for demonstration of added benefit over existing treatments and, if so, against which comparator? In this context, setting the regulatory bar unduly high might act as a deterrent for further innovation and drug development. Regulatory agencies are bound by their remit to not include cost considerations in their deliberations for authorisation. However, cost of treatments is a societal concern and will be a part of the Health Technology Assessment evaluation. In our view, while the regulatory hurdles should not be increased, there is the need for evaluation of clinical impact and added value including cost effectiveness of these agents where large datasets suggest a limited benefit. This may imply encouraging conduct of comparative benefit trials that include cost benefit evaluation.

DPP4i have had a surprisingly high uptake in the oral anti-diabetic market worldwide with an enthusiasm that can only be explained by marketing. In a period in which the costs to the National Health Services are increasing, we have to ask ourselves how long can we afford to pay for a small metabolic effect of newer drugs, such as DPP4i, whose annual cost is around £600.00/patient/year and the cost of treating diabetics is incremental annually [10]. New drugs are always needed for a patient-centred approach aimed to achieve a better control of glycaemia and reduce the risk of negative outcomes but the price of the new drugs should be related to their clinical benefit ensuring a cost-effective use of the medications.

## Conclusions

With increasing population of diabetics worldwide the cost of treating this disease is escalating and all health authorities are compelled to bring cost–effectiveness to the fore. Therefore, it will be important to consider what requires to be focus of introducing newer treatments for T2DM and whether a refocus of the objectives of large trials for newer class of treatment is necessary. A further point that needs consideration is how do we define benefits of newer class of agents such as the DDP4i that have only modest effect on glycaemic control and no effect on hard clinical end points.

Therefore, a better collaboration among all stakeholders is needed in order to develop studies with endpoints that will be both clinically meaningful and economically relevant in order to justify the increased costs of newer drugs that can be of benefit for patients with T2DM.

## Abbreviations

DPP4i: dipeptidyl peptidase 4 inhibitors
HbA1c: glycated haemoglobin
HF: heart failure
OADs: oral anti-diabetic drugs
STGL2i: sodium-glucose co-transporter 2
T2DM: type 2 diabetes mellitus
